# Comparison of diagnostic performance of radiologist- and AI-based assessments of T2-FLAIR mismatch sign and quantitative assessment using synthetic MRI in the differential diagnosis between astrocytoma, IDH-mutant and oligodendroglioma, IDH-mutant and 1p/19q-codeleted

**DOI:** 10.1007/s00234-024-03288-0

**Published:** 2024-01-15

**Authors:** Kazufumi Kikuchi, Osamu Togao, Koji Yamashita, Daichi Momosaka, Yoshitomo Kikuchi, Daisuke Kuga, Sangatsuda Yuhei, Yutaka Fujioka, Fumiya Narutomi, Makoto Obara, Koji Yoshimoto, Kousei Ishigami

**Affiliations:** 1 Department of Molecular Imaging and Diagnosis, Graduate School of Medical Sciences, Kyushu University, 3-1-1 Maidashi, Higashi-Ku, Fukuoka, 812-8582 Japan; 2 Department of Clinical Radiology, Graduate School of Medical Sciences, Kyushu University, 3-1-1 Maidashi, Higashi-Ku, Fukuoka, 812-8582 Japan; 3 Department of Neurosurgery, Graduate School of Medical Sciences, Kyushu University, 3-1-1 Maidashi, Higashi-Ku, Fukuoka, 812-8582 Japan; 4 Department of Anatomic Pathology, Pathological Sciences, Graduate School of Medical Sciences, Kyushu University, 3-1-1 Maidashi, Higashi-Ku, Fukuoka, 812-8582 Japan; 5 Philips Japan Ltd., 2-13-37, Konan, Minato-Ku, Tokyo, 108-8507 Japan

**Keywords:** Artificial intelligence, Convolutional neural network, Synthetic MRI, T2-FLAIR mismatch sign

## Abstract

**Purpose:**

This study aimed to compare assessments by radiologists, artificial intelligence (AI), and quantitative measurement using synthetic MRI (SyMRI) for differential diagnosis between astrocytoma, IDH-mutant and oligodendroglioma, and IDH-mutant and 1p/19q-codeleted and to identify the superior method.

**Methods:**

Thirty-three cases (men, 14; women, 19) comprising 19 astrocytomas and 14 oligodendrogliomas were evaluated. Four radiologists independently evaluated the presence of the T2-FLAIR mismatch sign. A 3D convolutional neural network (CNN) model was trained using 50 patients outside the test group (28 astrocytomas and 22 oligodendrogliomas) and transferred to evaluate the T2-FLAIR mismatch lesions in the test group. If the CNN labeled more than 50% of the T2-prolonged lesion area, the result was considered positive. The T1/T2-relaxation times and proton density (PD) derived from SyMRI were measured in both gliomas. Each quantitative parameter (T1, T2, and PD) was compared between gliomas using the Mann–Whitney *U*-test. Receiver-operating characteristic analysis was used to evaluate the diagnostic performance.

**Results:**

The mean sensitivity, specificity, and area under the curve (AUC) of radiologists vs. AI were 76.3% vs. 94.7%; 100% vs. 92.9%; and 0.880 vs. 0.938, respectively. The two types of diffuse gliomas could be differentiated using a cutoff value of 2290/128 ms for a combined 90^th^ percentile of T1 and 10^th^ percentile of T2 relaxation times with 94.4/100% sensitivity/specificity with an AUC of 0.981.

**Conclusion:**

Compared to the radiologists’ assessment using the T2-FLAIR mismatch sign, the AI and the SyMRI assessments increased both sensitivity and objectivity, resulting in improved diagnostic performance in differentiating gliomas.

**Supplementary Information:**

The online version contains supplementary material available at 10.1007/s00234-024-03288-0.

## Introduction

Isocitrate dehydrogenase (IDH) enzymes play a key role in glioma tumorigenesis [1]. A previous study revealed that IDH mutations were more frequently observed in diffuse low-grade gliomas, including astrocytomas and oligodendrogliomas [2]. The two types of gliomas share the same IDH mutation status, but their prognoses differ [3]. Oligodendrogliomas, IDH-mutant and 1p/19q-codeleted have a better prognosis and respond better to chemotherapy or radiotherapy than astrocytomas, IDH-mutant [4], while astrocytomas require more intensive treatment. Therefore, an accurate diagnosis is essential for effective patient management [5]. In 2017, Patel et al. [3] reported that astrocytoma, IDH-mutant exhibited the T2-FLAIR mismatch sign. Subsequently, numerous studies on the T2-FLAIR mismatch sign have been published [5–8]. The T2-FLAIR mismatch sign has a high specificity of 100% but a low sensitivity ranging from 12 to 51% in the diagnosis of astrocytoma, IDH-mutant [3, 7]. This is due to the strict criteria used to maintain high specificity and the wide range of the interobserver agreement, which makes it dependent on observer subjectivity, leading to significant interobserver variability [6].

Potential solutions to this problem include artificial intelligence (AI) modalities such as deep learning and quantitative approaches based on relaxation time measurements. In recent years, there has been considerable research regarding AI as an adjunct to imaging diagnostics, with some studies suggesting that it can outperform radiologists in certain diagnostic tasks [9–12]. Moreover, there are reports that the combination of AI-detected lesions and human assessment can lead to an even higher diagnostic accuracy [13, 14]. By eliminating subjective judgments and highlighting areas of T2-FLAIR mismatch, it is proposed that AI could be a valuable asset in this context. To the best of our knowledge, no previous studies on AI assessments of the T2-FLAIR mismatch sign have been reported.

Another solution might be a quantitative method that offers inherent objectivity. In addition to ensuring consistency, the capacity to make numerical judgments eliminates much of the subjectivity that can occasionally result in biases or errors in the interpretation of data. Previous studies have reported that measurement of relaxation time can improve sensitivity in T2-FLAIR mismatch lesions [5, 15].

Therefore, this study aimed to compare assessments by radiologists, artificial intelligence (AI), and quantitative measurement using synthetic MRI (SyMRI) for differential diagnosis between astrocytoma, IDH-mutant and oligodendroglioma, and IDH-mutant and 1p/19q-codeleted and to identify the superior method.

## Materials and methods

The institutional review board of our hospital approved this retrospective study, and the requirement for informed consent was waived. All methods were performed in accordance with the relevant guidelines and regulations.

### Patients

From June 2019 to December 2021, all patients at our institution who received a glioma diagnosis in a timely manner were eligible for this study. Inclusion criteria were (1) a diagnosis of IDH-mutant and/or 1p/19q-codeleted glioma based on the WHO 2021 classifications [1], (2) MRI scans performed within 2 weeks preceding surgery, and (3) available SyMRI evaluations. The exclusion criterion was image distortion, such as motion artifacts or noise. A total of 33 patients (men, 14; women, 19; age, 29–79 [median, 44] years) including 19 astrocytomas, IDH-mutant (men, 6; women, 13; age, 29–60 [median, 43] years; Grade 2, *n* = 12; Grade 3, *n* = 5; Grade 4, *n* = 2), and 14 oligodendrogliomas, IDH-mutant and 1p/19q-codeleted (men, 8; women, 6; age, 37–63 [median, 48] years; Grade 2, *n* = 10; Grade 3, *n* = 4) were included in the study.

For the machine learning training data, 50 patients other than those included in the study, treated at our hospital from June 2002 to December 2014, were selected. These comprised 28 astrocytomas, IDH-mutant (men, 20; women, 8; age, 20–52 [median, 33] years; Grade 2, *n* = 18; Grade 3, *n* = 10), and 22 oligodendrogliomas, IDH-mutant and 1p/19q-codeleted (men, 10; women, 12; age, 20–73 [median, 46] years; Grade 2, *n* = 13; Grade 3, *n* = 9).

### MR imaging

MR sequences used in this study were previously described [5]. All examinations were performed using a 3 T MR scanner (Ingenia 3.0 T CX; Philips Healthcare, Best, Netherlands) with a 15-channel head coil. Quantitative MRI was performed using the two-dimensional axial quantification of relaxation times and proton densities by the multi-echo acquisition of a saturation recovery using a turbo spin-echo readout (QRAPMASTER) pulse sequence with two echo times (TEs; 13 and 100 ms) and four delay times to generate eight real images and eight imaginary images [16]. The other parameters included repetition time (TR), 4831 ms; flip angle (FA), 90°; number of excitations (NEX), 1; sensitivity-encoding factor, 2.2; field of view (FOV), 230 × 189 (recon. 230 × 230) mm^2^; matrix, 512 × 512; echo-train length, 10; thickness/gap, 4.0/1.0 mm; 30 slices; and scan time 6 min 36 s. Quantification map acquisition was performed using SyMRI software (Version 19.0; SyMRI, Linköping, Sweden, https://syntheticmr.com/) [16]. Standard MR sequences (T1-weighted imaging [T1WI], T2WI, FLAIR, and contrast-enhanced T1WI) were also obtained. The sequence parameters of the two-dimensional axial T2WI and FLAIR sequences were as follows: T2WI—TR/TE, 3000/80 ms; FA, 90°; NEX, 1; FOV, 230 × 230 mm^2^; matrix, 512 × 375 (recon. 512 × 512); echo-training length, 15; thickness/gap, 5.0/1.0 mm; 22 slices; and scan time, 2 min 36 s, and FLAIR—TR/TE/TI, 10,000/120/2700 ms; FA, 180°; NEX, 1; FOV, 230 × 207 (recon. 230 × 230) mm^2^; matrix, 320 × 228 (recon. 512 × 512); echo-training length, 27; thickness/gap, 5.0/1.0 mm; 22 slices; and scan time, 3 min.

### Convolutional neural network model architecture

**Fig. 1:**
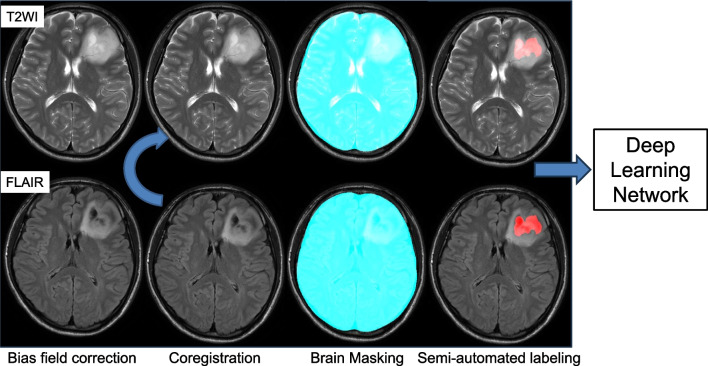
Image-preprocessing pipeline. (1) Bias field correction was applied to T2WI and FLAIR images, (2) FLAIR images were coregistered with T2WI images, (3) brain masking was created on T2WI images and propagated to the registered FLAIR images, and (4) labels were drawn using the semi-automated contouring tool

U-net and DeepMedic are widely used as convolutional neural networks (CNNs) in AI research. As for this study, DeepMedic was chosen because it has been reported to outperform U-net in intracranial atherosclerotic diseases [17]. We applied the DeepMedic network developed by Kamnitsas et al. [18], which is a multi-scale 3D CNN, to assess the T2-FLAIR mismatch lesion. In order to create a large receptive field for the final classification while retaining a low computational cost, this design comprises 11 layers and 2 parallel convolutional pathways that process the input at various scales. This architecture uses 3^3^ kernels, which are fewer than the usual 5^3^ kernels, to convolve quickly and minimize the weight. With these small kernels, deep network variants can be designed efficiently by reducing the number of multiplications and trainable parameters for each element. To incorporate both local and more general contextual information into the 3D CNN, a down-sampled second pathway was introduced. In the first pathway, the structure’s specific local appearance is recorded, whereas, in the second pathway, higher-level information like the structure’s location in the brain is learned [18]. The identification of the T2-FLAIR mismatch lesion as ground truth was manually determined by a certificated radiologist (K.K., with 16 years of experience in diagnostic radiology) who knew the pathological information of patients with diffuse glioma. Figure 1 shows the preprocessing pipeline using IntelliSpace Discovery (version 3, Philips Healthcare, Best, Netherlands). Both images of T2WI and FLAIR were bias-field-corrected. Then, in the second step, the corrected FLAIR images were coregistered to the reference space defined by the T2WI. Third, a brain mask was computed and applied to obtain skull-stripped images. Finally, labels were drawn using the semi-automated contouring tool. For detecting the T2-FLAIR mismatch lesion, the deep learning model (DeepMedic) was applied to the preprocessed data.

### T2-FLAIR mismatch evaluation and statistical analysis

#### Radiologist assessment

Four board-certified neuroradiologists (with 23, 21, 10, and 8 years of experience) were blinded to the patient information of the evaluated T2-FLAIR mismatch sign. The T2-FLAIR mismatch sign was defined by the presence of two distinct MRI features as follows [3, 6]: (1) The tumor displayed a complete or nearly complete and nearly homogeneous hyperintense signal on T2WI and (2) the tumor displayed a relatively hypointense signal on the FLAIR sequence except for a hyperintense peripheral rim. Further, Jain et al. [6] introduced additional imaging features aiding in the accurate identification of the T2-FLAIR mismatch sign: (3) Necrotic cavities do not represent the T2-FLAIR mismatch sign; small cysts do not meet the criteria for the T2-FLAIR mismatch sign. (4) The T2-FLAIR mismatch lesion is typically accompanied by little or no contrast enhancement. (5) The degree of FLAIR signal suppression could be inhomogeneous within the tumor. (6) Common imaging correlates include homogeneous hypointensity on non-contrast T1WI, markedly elevated apparent diffusion coefficient values, low blood volume on perfusion maps, and diffuse hypodensity on CT. After independent data collection, the interreader agreement was calculated. Four radiologists read both the T2WI and FLAIR images based on whether the T2-FLAIR mismatch sign was present or absent. The sensitivity, specificity, positive predictive value (PPV), negative predictive value (NPV), and accuracy were calculated. In the radiologist’s evaluation, the interrater agreement for the T2-FLAIR mismatch sign among the four observers was evaluated using Fleiss’s kappa coefficient [19]. The kappa value was interpreted as follows: almost perfect agreement, 1.00–0.81; substantial agreement, 0.80–0.61; moderate agreement, 0.60–0.41; fair agreement, 0.40–0.21; slight agreement, 0.20–0.01; and poor agreement, < 0 [20].

#### Artificial intelligence assessment based on the convolutional neural network

If the CNN labeled more than 50% of the T2-prolonged lesion area, it was considered positive, defining the presence of the T2-FLAIR mismatch sign. While there is a method using the Dice coefficient, this study is binary in nature; therefore, we simply determined it based on appearance.

#### Quantitative assessment using synthetic MRI

The DICOM data of the T1 and T2 relaxation times and proton density map were extracted by SyMRI software (version 19.0; SyMRI, Linköping, Sweden, https://syntheticmr.com/) [16]. We used a single maximum section of each tumor for the regions of interest (ROI) analysis on the T2-prolonged region in the tumor using an ImageJ plugin (ImageJ/Fiji; version 2.0.0-rc-59/1.51 k, National Institutes of Health, Bethesda, MD). The maximum section of the tumor was visually determined as the largest orthogonal cross-product of the tumor on the axial T2WI/FLAIR [5]. Using the ROI manager tool of ImageJ/Fiji, the ROI mask from the T2-prolonged region on conventional T2WI scans was copied and placed on each parameter map (T1 and T2 relaxations and proton density maps) to obtain pixel-by-pixel values for the histogram analyses. The 10th, 25th, 50th, 75th, and 90th percentiles and the mean, skewness, and kurtosis of each parameter were recorded from the histograms. Each parameter (i.e., T1 and T2 relaxation times and proton density) was compared between astrocytomas, IDH-mutant and oligodendrogliomas, IDH-mutant and 1p/19q-codeleted using the Mann–Whitney *U*-test. The diagnostic performance of each parameter was evaluated using a receiver-operating characteristic curve analysis.

All statistical analyses were performed using commercial software programs (JMP, version 15.0.0; SAS Institute, Cary, NC, USA; Prism 7.0, GraphPad Software, La Jolla, CA, USA). *P* < 0.05 was considered statistically significant.

## Results

### Radiologist and artificial intelligence assessments

Table 1 shows the results from the four radiologists and AI. The mean sensitivity, specificity, accuracy, PPV, NPV, and the area under the curve (AUC) of radiologists vs. AI were 76.3% vs. 94.7%, 100% vs. 92.9%, 86.4% vs. 93.9%, 100% vs. 94.7%, 76.0% vs. 92.9%, and 0.880 vs. 0.938, respectively. An almost perfect interrater agreement was observed (kappa coefficient = 0.88).

**Table 1:** Radiologist and artificial intelligence assessment of T2-FLAIR mismatch sign

	Reader 1	Reader 2	Reader 3	Reader 4	Average*	AI
Sensitivity (%)	68.4	73.7	84.2	79.0	76.3	94.7
Specificity (%)	100.0	100.0	100.0	100.0	100.0	92.9
Accuracy (%)	81.8	84.9	90.9	87.9	86.4	93.9
PPV (%)	100.0	100.0	100.0	100.0	100.0	94.7
NPV (%)	70.0	73.7	82.4	77.8	76.0	92.9
AUC	0.850	0.868	0.912	0.889	0.880	0.938

*AI* artificial intelligence, *AUC* area under the curve, *NPV* negative predictive value, *PPV* positive predictive value

^*^The kappa coefficient among four radiologists was 0.88

### Quantitative assessment using synthetic MRI

**Fig. 2:**
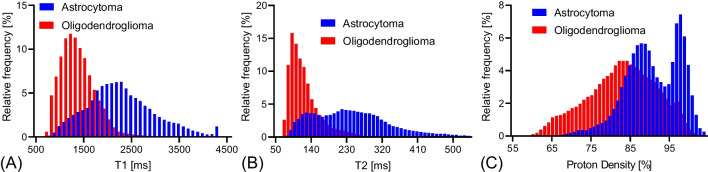
Histograms of T1 and T2 relaxation times and proton density (PD) between astrocytomas, IDH-mutant and oligodendrogliomas, IDH-mutant and 1p/19q-codeleted. All parameters (T1 and T2 relaxation times and PD) in astrocytomas exhibit a slight rightward shift relative to those in oligodendrogliomas

Figure 2 and Supplementary Table S1 show the histograms of each parameter over all the pixels in the tumor ROIs. T1 and T2 relaxation times and proton densities from the astrocytomas all exhibited a slight rightward shift relative to those from the oligodendrogliomas. T1 and T2 relaxation times and proton densities were larger for astrocytomas than for oligodendrogliomas (median values, 95% confidence intervals, and *p*-values—2503 (1967–2891) vs. 1385 (1119–1783) ms, *p* < 0.0001 for mean T1 relaxation time; 259 (214–343) vs. 121 (94–143) ms, *p* < 0.0001 for mean T2 relaxation time; and 94.9% (89.2–96.7%) vs. 85.2% (76.8–88.3%), *p* < 0.0001 for mean proton density, respectively). There were also significant differences in the 10–90th percentiles for T1 and T2 relaxation times and proton densities (all *p* < 0.05). Table 2 and Supplementary Table S2 show the diagnostic performance in differentiating the two glioma groups; the most useful values of each parameter are shown in Table 2. The two types of diffuse gliomas could be differentiated using a cutoff value of 2290/128 ms for a combined 90th percentile of T1 and 10th percentile of T2 relaxation times with 94.4% sensitivity, 100% specificity, 96.9% accuracy, 100% PPV, and 93.3% NPV, with an AUC of 0.981.

**Table 2:** Diagnostic performance of parameters in differentiating between astrocytoma, IDH-mutant and oligodendroglioma, and IDH-mutant and 1p/19q-codeleted

Parameters	Sensitivity (%)	Specificity (%)	Accuracy (%)	PPV (%)	NPV (%)	Cutoff	AUC
T1 [ms]							
75^th^ percentile	84.2	100.0	90.9	100.0	82.4	2107	0.966
90^th^ percentile	89.5	92.9	90.9	94.4	86.7	2290	0.966
T2 [ms]							
10^th^ percentile	100.0	85.7	93.9	90.5	100.0	99	0.977
PD [%]							
10^th^ percentile	79.0	100.0	87.9	100.0	77.8	83.4	0.917
Combined 90^th^ T1 and 10^th^ T2	94.4	100.0	96.9	100.0	93.3	2290/128	0.981

*AUC* area under the curve, *IDH* isocitrate dehydrogenase, *NPV* negative predictive value, *PD* proton density, *PPV* positive predictive value

**Fig. 3:**
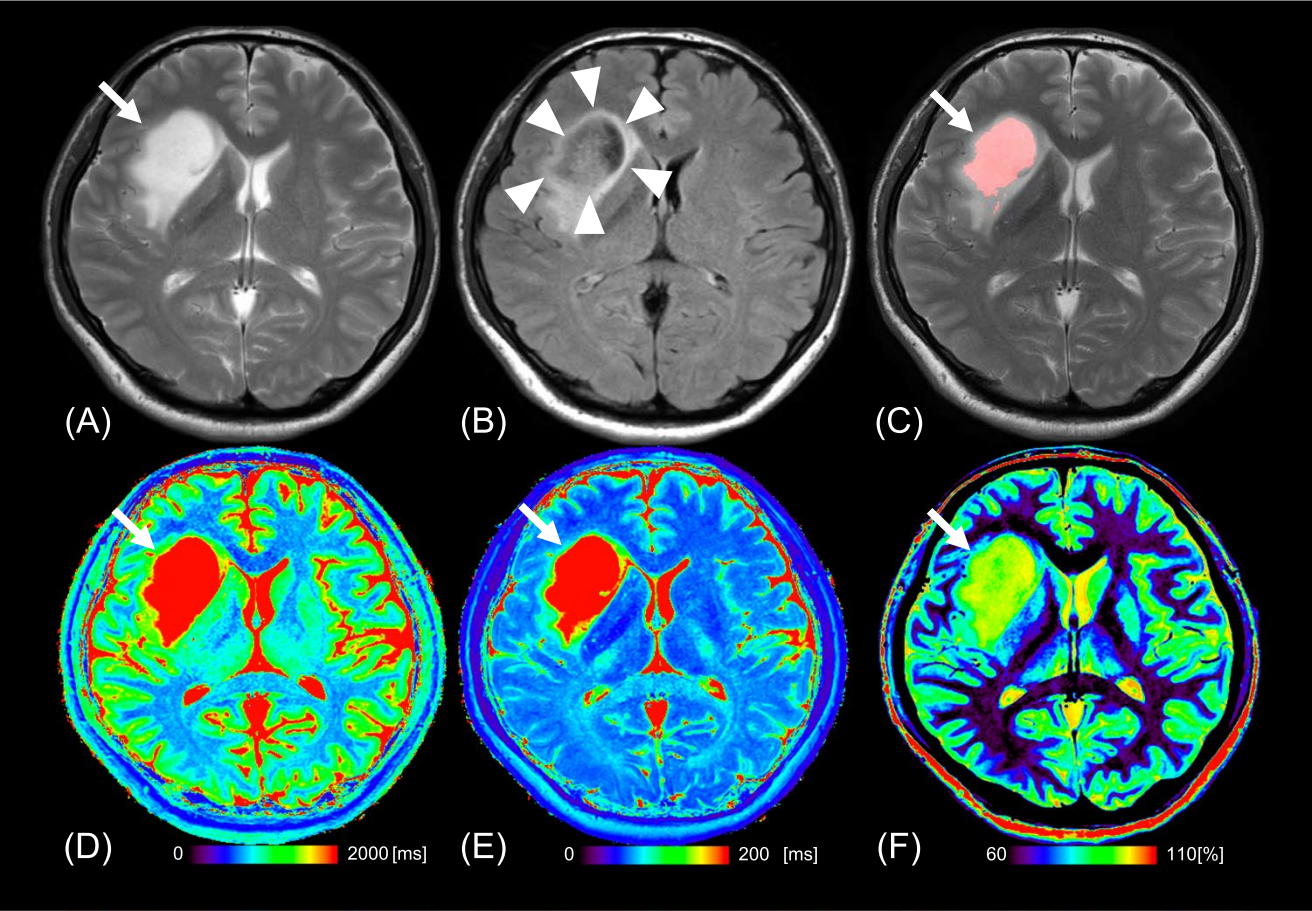
Images from a 39-year-old man with astrocytoma, IDH-mutant (WHO Grade 2). **a** T2WI shows a homogeneous T2-prolonged mass in the right insula (arrow). **b** FLAIR shows partial signal suppression, indicating a T2-FLAIR mismatch sign (arrowheads). **c** Our artificial intelligence correctly detects this T2-FLAIR mismatch lesion (arrow). T1 (**d**), T2 (**e**), and relaxation time and proton density (**f**) maps derived from SyMRI show T1 (2891 ms*) and T2 (375 ms*) relaxation time prolongations and increased PD (96.1%*) (arrows) in the tumor Asterisks (*) beside the values in the caption indicates that each value is expressed as the mean

**Fig. 4:**
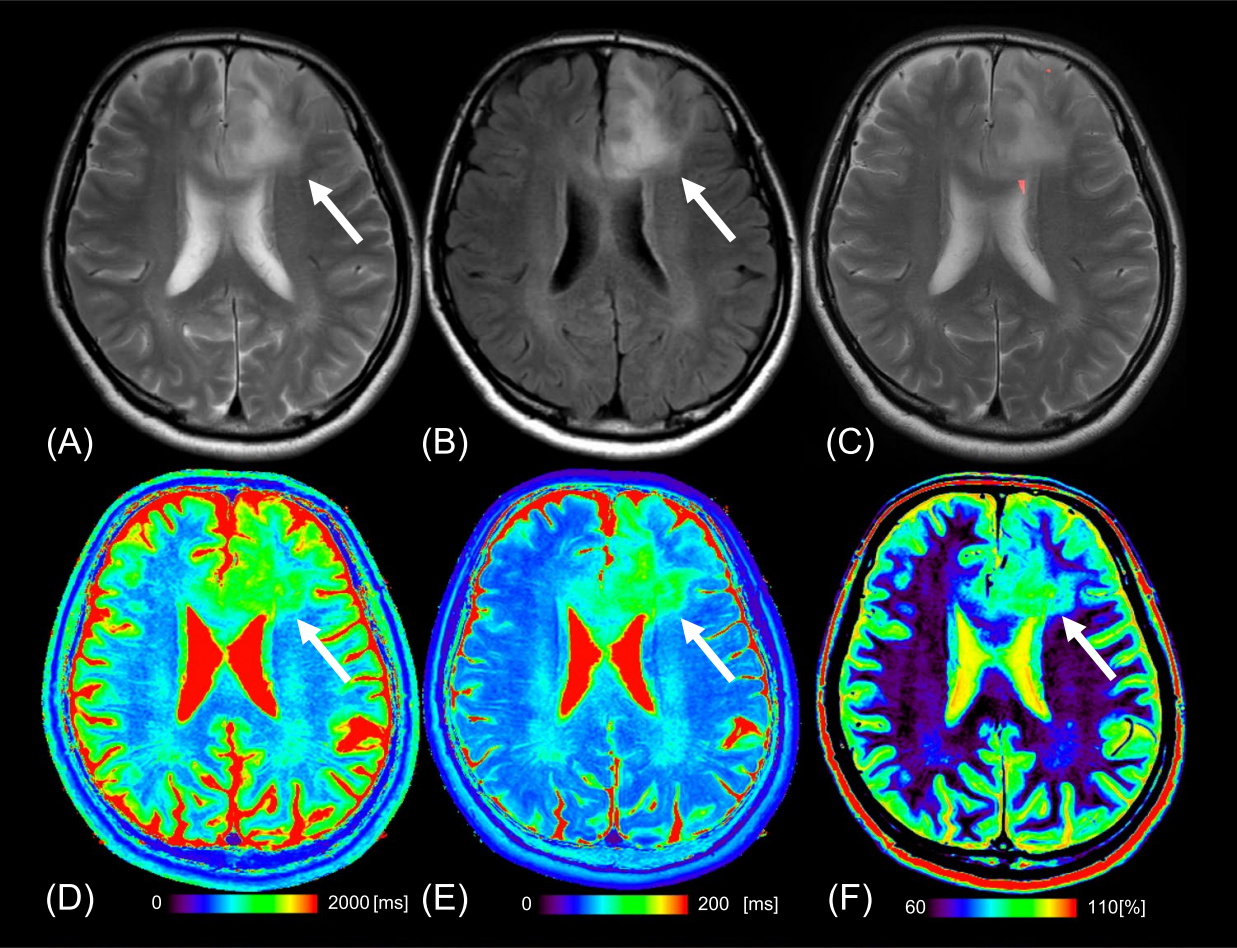
Images from a 54-year-old man with oligodendroglioma, IDH-mutant and 1p/19q-codeleted (WHO Grade 2). **a** T2WI shows a heterogeneous, poorly circumscribed mass in the bilateral frontal lobes (arrow). **b** FLAIR shows no signal suppression in the tumor, indicating no T2-FLAIR mismatch sign (arrow). **c** Our artificial intelligence does not correctly show this T2-FLAIR mismatch lesion. T1 (**d**), T2 (**e**), and relaxation time and proton density (**f**) maps derived from SyMRI show mild T1 (1783 ms*) and T2 (121 ms*) relaxation time prolongations and mildly increased PD (81.7%*) (arrows) in the tumor. Asterisks (*) beside the values in the caption indicate that each value is expressed as the mean

Figures 3 and 4 show representative images of patients with astrocytoma and oligodendroglioma, respectively.

## Discussion

We found that both AI and SyMRI improved the sensitivity of T2-FLAIR mismatch lesions as well as the diagnostic performance in the differential diagnosis between astrocytoma, IDH-mutant and oligodendroglioma, and IDH-mutant and 1p/19q-codeleted compared to radiologists in this study. The determination of the T2-FLAIR mismatch sign is subjective, resulting in variability. This study showed that by eliminating subjectivity, sensitivity was improved. The use of AI offers a distinct advantage regarding versatility; once a model is refined and completed, it can be deployed across different institutions or settings, ensuring widespread applicability. This universal adaptability is a compelling strength of AI-driven solutions. On the other hand, the quantitative assessment by SyMRI has unique advantages. The ability to evaluate data numerically provides inherent objectivity. This ability to provide numerical assessments not only ensures consistency but also removes much of the human subjectivity that can sometimes lead to inconsistencies or biases in data interpretation. Therefore, while AI offers flexibility and adaptability, tools like SyMRI offer rigorous, objective analysis. The present study method is expected to increase the accuracy of preoperative brain tumor diagnosis. Because the AI model can be used for transfer learning, PACS equipped with AI applications could find widespread use. On the other hand, relaxation time can also be measured with conventional MRI using the multi-echo method instead of SyMRI.

Previous studies have provided valuable insight into radiologists’ assessment of the T2-FLAIR mismatch sign. Sensitivity in these studies has been found to range from 22 to 57% [3, 7]. The interrater agreement among radiologists has also shown a considerable range, with *κ* (kappa coefficient) values ranging from 0.38 to 0.88 [8]. Notably, Jain et al. emphasized the importance of applying strict criteria to maintain a high level of specificity, even though this is often at the expense of reduced sensitivity [6]. Our research findings are consistent with the trends observed in these earlier studies. The relatively low interrater agreement among radiologists is likely due to the binary scoring system that is commonly used. This system may not adequately capture the nuances of the T2-FLAIR mismatch sign, as subtle variations in imaging characteristics may lead to different interpretations by different readers [5, 8].

The high sensitivity of AI in detecting T2-FLAIR mismatch lesions is believed to be due to its retrospective learning approach, where it learns to identify these lesions after having access to pathology results. In other words, it operates in a “cheat mode” that enables it to achieve greater sensitivity. To our knowledge, there are no previous studies that have attempted to detect T2-FLAIR mismatch lesions using AI. We used the DeepMedic network proposed by Kamnitsas et al. [18], which is efficient in learning even with a small dataset. Because this model employs a multi-scale approach to capture information at different levels of detail, it can still extract useful features from the data even when the dataset is small, thereby optimizing the available information. Kikuchi et al. reported that they used DeepMedic trained on 50 patients with 165 lesions to detect brain metastases [9]. Although the amount of training data is smaller than in previous studies (number of training cases/lesions = 188–469/917–1149) [11, 12], DeepMedic demonstrates a detection sensitivity for brain metastases that is comparable with that of radiologists [9]. This suggests that DeepMedic can effectively learn from a limited number of cases.

SyMRI has been shown to be valuable in increasing the sensitivity for differential diagnosis between astrocytoma, IDH-mutant and oligodendroglioma, IDH-mutant and 1p/19q-codeleted. Previous studies have also highlighted the benefits of using quantitative relaxation time assessments in the context of T2-FLAIR mismatch lesions, which have consistently resulted in increased sensitivity [5, 15]. The results of the present study are consistent with these previous research findings and underscore the utility of such quantitative assessments. In this study, an increase in sensitivity was observed in the measurement of relaxation time; however, the specificity of the single parameter decreased slightly. Previous research on the pathologic evaluation of the T2-FLAIR mismatch sign has shown that regions with T2-FLAIR mismatch have microcystic changes, leading to prolongation of relaxation time due to increased fluid components [21]. Differential diagnosis is difficult when astrocytoma, IDH-mutant presents without microcystic change because of the lack of prolonged relaxation time. Nevertheless, the combination with the relaxation parameters of T1 and T2 exhibited improved diagnostic performance. Based on this result, it can be concluded that the combination of T1 and T2 relaxation times provides a better understanding of the tissue structure within the T2-FLAIR mismatch lesions.

This study has several limitations. First, our study comprised postoperative cases and had a small sample size since IDH-mutant-type gliomas are relatively rare and there are limitations to collecting cases at a single center. Because an AI-based classification study requires a large sample size, this may raise concerns about the reliability of the results; therefore, further studies may require multicenter validation. Second, we did not assess IDH-wild-type astrocytomas in our investigation. A follow-up study with patients with IDH-wild-type astrocytomas would be useful. Third, we did not include the whole tumor volume for the histogram analysis in the quantitative evaluation. Instead, we used the maximum section of the tumor, with its boundary defined by the hyperintensity on T2WI. However, only the largest region of the tumor was used in the previous research on the T2-FLAIR mismatch sign; whole-volume histogram analysis was not conducted. Since the T2-FLAIR mismatch sign criteria are designed to retain high specificity rather than boost sensitivity, by using these tight criteria, a simple evaluation based on the maximum-sized slice of the tumor may be sufficient. Although a quantitative whole-tumor analysis would probably yield results that differ from those currently presented, it is likely that astrocytomas, IDH-mutant would have exhibited longer T1 and T2 values than oligodendrogliomas, IDH-mutant and 1p/19q-codeleted.

In conclusion, compared to radiologists’ assessments using the T2-FLAIR mismatch sign, the AI and the SyMRI assessments increased both sensitivity and objectivity, resulting in improved diagnostic performance in differentiating astrocytomas, IDH-mutant from oligodendrogliomas, IDH-mutant and 1p/19q-codeleted.

### Supplementary Information

Below is the link to the electronic supplementary material.

Supplementary file1 (DOCX 52 KB)

## Data Availability

The datasets generated during the current study are not available because of our institutional policy.
