# Supplementary material for: Comparison of diagnostic performance of radiologist- and AI-based assessments of T2-FLAIR mismatch sign and quantitative assessment using synthetic MRI in the differential diagnosis between astrocytoma, IDH-mutant and oligodendroglioma, IDH-mutant and 1p/19q-codeleted
Source: Neuroradiology. 2024 Jan 15;66(3):333–41. doi: 10.1007/s00234-024-03288-0 (PMC10859342; doi:10.1007/s00234-024-03288-0)
Supplement: Supplementary file 1 — Supplementary file1 (DOCX 52 KB) [file 234_2024_3288_MOESM1_ESM.docx]

**Comparison of diagnostic performance of radiologist- and AI-based assessment of T2-FLAIR mismatch sign and quantitative assessment using synthetic MRI in the differential diagnosis between astrocytoma, IDH-mutant and oligodendroglioma, IDH-mutant and 1p/19q-codeleted**

**Data, Materials, and/or Code availability:** The datasets generated during the current study are not available because of our institutional policy.

**Supplementary Table S1. Comparison of the parameters between astrocytoma, IDH-mutant vs. oligodendroglioma, IDH-mutant and 1p/19q-codeleted**

| **Parameter** | **Astrocytoma, IDH-mutant** | **Oligodendroglioma, IDH-mutant and 1p/19q-codeleted** | **^a^*P*-value** |
| --- | --- | --- | --- |
| T1 [ms] |  |  |  |
| 10^th^ percentile | 2006 (1531–2096) | 1112 (845–1308) | < 0.0001 |
| 25^th^ percentile | 2184 (1719–2443) | 1208 (953–1492) | < 0.0001 |
| 50^th^ percentile | 2406 (1931–2904) | 1350 (1113–1745) | < 0.0001 |
| 75^th^ percentile | 2753 (2149–3348) | 1579 (1269–1959) | < 0.0001 |
| 90^th^ percentile | 2955 (2372–3688) | 1731 (1399–2101) | < 0.0001 |
| Mean | 2503 (1967–2891) | 1385 (1119–1783) | < 0.0001 |
| Skewness | 0.22 (–0.10–0.88) | 0.89 (0.51–1.06) | 0.0288 |
| Kurtosis | 0.37 (–0.37–1.79) | 1.59 (0.45–7.26) | 0.0383 |
| T2 [ms] |  |  |  |
| 10^th^ percentile | 176 (132–208) | 83 (78–98) | < 0.0001 |
| 25^th^ percentile | 196 (161–246) | 97 (86–115) | < 0.0001 |
| 50^th^ percentile | 248 (203–299) | 114 (93–135) | < 0.0001 |
| 75^th^ percentile | 312 (230–399) | 134 (100–163) | < 0.0001 |
| 90^th^ percentile | 353 (272–521) | 160 (112–179) | < 0.0001 |
| Mean | 259 (214–343) | 121 (94–143) | < 0.0001 |
| Skewness | 0.91 (0.56–1.84) | 1.03 (0.61–2.65) | 0.6021 |
| Kurtosis | 1.46 (0.15–5.72) | 1.76 (0.10–9.91) | 0.9570 |
| PD [%] |  |  |  |
| 10^th^ percentile | 88.4 (83.4–92.2) | 77.5 (70.1–81.5) | < 0.0001 |
| 25^th^ percentile | 90.6 (86.8–95.5) | 81.7 (73.2–84.3) | < 0.0001 |
| 50^th^ percentile | 95.8 (89.0–97.4) | 85.3 (77.1–88.7) | < 0.0001 |
| 75^th^ percentile | 98.0 (91.2–99.0) | 89.3 (83.1–92.9) | 0.0002 |
| 90^th^ percentile | 99.3 (93.8–101.4) | 92.7 (87.3–96.2) | 0.0003 |
| Mean | 94.9 (89.2–96.7) | 85.2 (76.8–88.3) | < 0.0001 |
| Skewness | –1.40 (–2.34– –0.26) | –0.47 (–0.91–0.09) | 0.0461 |
| Kurtosis | 5.34 (1.53–12.34) | 0.67 (–0.72–1.87) | 0.0014 |

Data are expressed as median values and 95% confidence intervals.

*PD*, proton density

^a^Mann–Whitney U-test

**Supplementary Table 2.** **Diagnostic performance of the parameters differentiating between astrocytoma, IDH-mutant and oligodendroglioma, IDH-mutant and 1p/19q-codeleted**

| **Parameter** | **Sensitivity [%]** | **Specificity [%]** | **Accuracy [%]** | **PPV [%]** | **NPV [%]** | **Cutoff** | **AUC** |
| --- | --- | --- | --- | --- | --- | --- | --- |
| T1 [ms] |  |  |  |  |  |  |  |
| 10^th^ percentile | 84.2 | 100.0 | 90.9 | 100.0 | 82.4 | 1527 | 0.940 |
| 25^th^ percentile | 84.2 | 100.0 | 90.9 | 100.0 | 82.4 | 1683 | 0.944 |
| 50^th^ percentile | 89.5 | 100.0 | 93.9 | 100.0 | 87.5 | 1861 | 0.959 |
| 75^th^ percentile | 84.2 | 100.0 | 90.9 | 100.0 | 82.4 | 2107 | 0.966 |
| 90^th^ percentile | 89.5 | 92.9 | 90.9 | 94.4 | 86.7 | 2290 | 0.966 |
| Mean | 89.5 | 100.0 | 93.9 | 100.0 | 87.5 | 1882 | 0.959 |
| Skewness | 63.2 | 92.9 | 75.8 | 92.3 | 65.0 | 0.38 | 0.726 |
| Kurtosis | 57.9 | 85.7 | 69.7 | 84.6 | 60.0 | 0.40 | 0.714 |
| T2 [ms] |  |  |  |  |  |  |  |
| 10^th^ percentile | 100.0 | 85.7 | 93.9 | 90.5 | 100.0 | 99 | 0.977 |
| 25^th^ percentile | 84.2 | 100.0 | 90.9 | 100.0 | 82.4 | 146 | 0.970 |
| 50^th^ percentile | 94.7 | 85.7 | 90.9 | 90.0 | 92.3 | 148 | 0.966 |
| 75^th^ percentile | 94.7 | 85.7 | 90.9 | 90.0 | 92.3 | 210 | 0.951 |
| 90^th^ percentile | 94.7 | 85.7 | 90.9 | 90.0 | 92.3 | 230 | 0.914 |
| Mean | 94.7 | 92.9 | 93.9 | 94.7 | 92.9 | 178 | 0.970 |
| Skewness | 52.6 | 71.4 | 60.6 | 71.4 | 52.6 | 0.91 | 0.556 |
| Kurtosis | 42.1 | 78.6 | 57.6 | 72.7 | 50.0 | 0.40 | 0.508 |
| PD [%] |  |  |  |  |  |  |  |
| 10^th^ percentile | 79.0 | 100.0 | 87.9 | 100.0 | 77.8 | 83.4 | 0.917 |
| 25^th^ percentile | 79.0 | 92.9 | 84.9 | 93.8 | 76.5 | 86.8 | 0.904 |
| 50^th^ percentile | 79.0 | 85.7 | 81.8 | 88.2 | 75.0 | 89.0 | 0.880 |
| 75^th^ percentile | 68.4 | 100.0 | 81.8 | 100.0 | 70.0 | 96.9 | 0.861 |
| 90^th^ percentile | 73.7 | 92.9 | 81.8 | 93.3 | 72.2 | 96.9 | 0.857 |
| Mean | 73.7 | 92.9 | 81.8 | 93.3 | 72.2 | 90.2 | 0.887 |
| Skewness | 63.2 | 92.9 | 75.8 | 92.3 | 65.0 | -1.24 | 0.707 |
| Kurtosis | 73.7 | 92.9 | 81.8 | 93.3 | 72.2 | 2.20 | 0.820 |
|  |  |  |  |  |  |  |  |
| Combined 90^th^ T1 and 10^th^ T2 | 94.4 | 100.0 | 96.9 | 100.0 | 93.3 | 2290/128 | 0.981 |

*AUC*, area under the curve; *NPV*, negative predictive value; *PD*, proton density; *PPV*, positive predictive value
